# Concurrence of Meningomyelocele and Salt-Wasting Congenital Adrenal Hyperplasia due to 21-Hydroxylase Deficiency

**DOI:** 10.1155/2015/196374

**Published:** 2015-01-19

**Authors:** Heves Kırmızıbekmez, Rahime Gül Yesiltepe Mutlu, Serdar Moralıoğlu, Ahmet Tellioğlu, Ayşenur Cerrah Celayir

**Affiliations:** ^1^Pediatric Endocrinology, Zeynep Kamil Obstetrics and Pediatrics Education and Research Hospital, 34668 Istanbul, Turkey; ^2^Pediatric Surgery, Zeynep Kamil Obstetrics and Pediatrics Education and Research Hospital, 34668 Istanbul, Turkey; ^3^Department of Pediatrics, Zeynep Kamil Obstetrics and Pediatrics Education and Research Hospital, 34668 Istanbul, Turkey

## Abstract

Congenital adrenal hyperplasia (CAH) is a group of inherited defects of cortisol biosynthesis. A case of classical CAH due to 21-hydroxylase deficiency (21-OHD) with early onset of salt waste and concurrence of meningomyelocele (MMC) was presented here. The management of salt-wasting crisis which is complicated by a postrenal dysfunction due to neurogenic bladder was described. Possible reasons of growth retardation in the one-year follow-up period were discussed. A significant regression of the phallus with proper medical treatment was also mentioned.

## 1. Introduction

Congenital adrenal hyperplasia (CAH) is a group of autosomal recessive disorders characterized by impaired cortisol synthesis leading to excessive corticotrophin stimulation of the adrenal cortex. More than 90% of CAH are caused by 21-hydroxylase deficiency (21-OHD), found in 1 : 10 000 to 1 : 15 000 live births. Potentially lethal adrenal insufficiency is characteristic of two-thirds to three-quarters of patients with the classical salt-wasting form of 21-OHD [1]. Accumulation of cortisol precursors that are diverted to androgens is the cause of virilisation in a female fetus. CAH is the most common cause of 46, XX Disorders of Sex Development (DSD). This case report defines the management of a patient with salt-wasting CAH and neurogenic bladder.

The most common cause of neurogenic bladder in children is neurospinal dysraphism [2]. MMC is the most common neural tube defect. It is characterized by a cleft in the vertebral column, with a corresponding defect in the skin so that the meninges and spinal cord are exposed. Nearly all patients with MMC have bladder dysfunction. This may adversely affect urinary continence and quality of life and can also lead to progressive deterioration of the upper urinary tract and chronic renal disease [3].

## 2. Case Report

A newborn was referred to our hospital for ambiguous genitalia on the first day of the birth. The weight was 3000 gr, the length was 50 cm, and the head circumference was 37 cm. The baby was born by caesarean section at the 37th week of the gestation. The parents were both healthy and no history of prenatal complication or medication was present. Parents were said to be nonconsanguineous; however, their family roots came from the same village.

**Figure 1:**
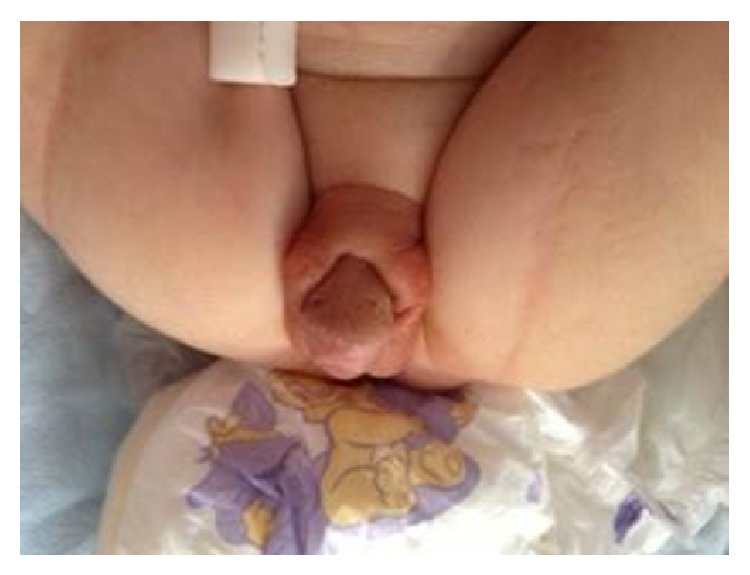
The external genitalia appeared to be significantly virilised (Prader stage 3).

**Figure 2:**
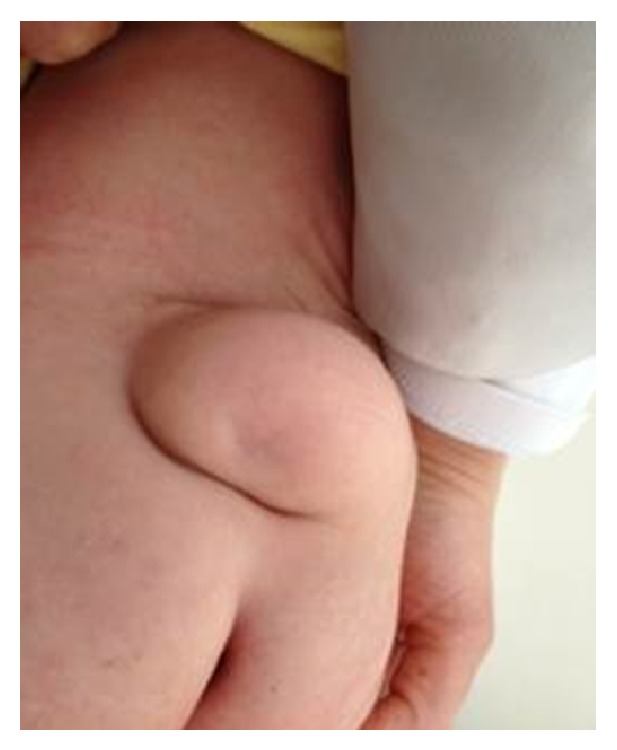
The meningomyelocele in the sacral area.

Overall, the patient appeared healthy and the vital findings were normal and no dysmorphic finding was present. However, a MMC was found in the sacral area. The inspection of external genitalia revealed a phallus of 2.5 × 2 cm in size, no palpable gonad, and a single orifice at the base of the phallus (Figures 1 and 2). Bilateral ovaries, fallopian tubes, and a uterus were present in the pelvic ultrasonography. The initial findings were suggesting 46, XX DSD, most probably CAH.

The patient's general status, vitals, feeding tolerance, glucose, and electrolyte levels were followed up carefully. On the 4th day, hypoactivity, poor feeding, and vomiting commenced. Electrolytes were suggesting salt waste. We stopped enteral feeding and before starting treatment we obtained blood samples for hormone tests. Fluid and electrolyte replacement began with saline and dextrose solutions (150 mL/kg/day, 100 mEq/L Na, and 10% dextrose). At the time, only methyl-prednisolone was available for an intravenous injection. At the beginning of the treatment we administered 5 mg of methyl-prednisolone and continued with divided equal doses every eight hours. Fludrocortisone was given (0.2 mg/day) for mineralocorticoid replacement. We also had to give 1 mg/day of table salt for a few days. On the 5th day, the patient's general appearance was better, vitals were normal, vomiting stopped, and electrolytes were improving. On the 6th day, feeding was well tolerated and electrolytes were normal. The amount of intravenous fluid and the dose of glucocorticoid treatment were reduced and continued with hydrocortisone tablets. Although the management of adrenal insufficiency was working properly, oliguria and declined renal functions occurred on the 7th day. Urine output was below 0.5 mL/kg/hr and creatinine level was 1.6 mg/dL. Physical examination revealed a “globe vesical,” and urine output and renal functions improved after urinary catheterization.

The androgen levels were high as expected (Table 1). Adrenocorticotrophic hormone (ACTH) level was confirming that the baby had primary adrenal insufficiency and renin level had just started to increase due to the initiation of salt waste. The results of hormone studies and karyotype analyses (46, XX) further supported the diagnosis of salt-wasting CAH. 21OHD was confirmed by genetic analyses. A homozygous deletion of E1–E3 30 kb (P30L-I2G-8 bp del) in the CYP21 gene was detected. It was a previously known mutation, which has a genotype-phenotype correlation with salt-wasting CAH.

**Figure 3:**
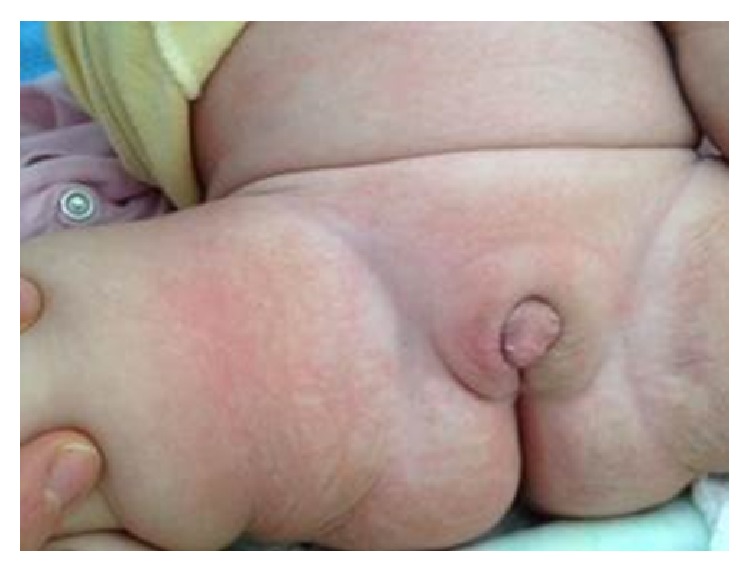
Regression of the phallus at the 7th month of the hormone replacement treatment (constructive operation has not yet been performed).

**Figure 4:**
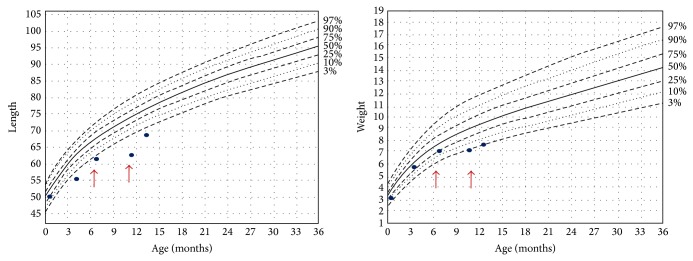
Growth retardation was occurring especially between 7th and 11th months.

Committee on Disorders of Sex Development agreed on the decision to raise the patient as a girl and planned to perform the necessary constructive operations for female genitalia. The phallus was significantly regressed at the 7th month of the hormone replacement treatment. It was 1.5 × 1 cm in size, and a constructive operation has not yet been performed (Figure 3). Between 7th and 11th months, she was hospitalized four times because of urinary tract infection. The parents were asked to give hydrocortisone in double doses in case of fever and other systemic infection findings. Growth retardation was occurring, especially in this period (Figure 4). Fortunately, neuromotor development was normal and she could walk by the time she is 13 months old. She is also under supervision of a neurosurgeon, a pediatric urologist, and a pediatric nephrologist.

## 3. Discussion

Congenital adrenal hyperplasia is a group of inherited defects of cortisol biosynthesis. The condition can be classified into “salt-wasting,” “simple virilising,” and “nonclassical” forms. These are not different diseases but represent points on a spectrum of disease severity directly related to the degree of enzymatic compromise conferred by a given genetic defect [1]. Females with the classical form present with genital ambiguity. Males with the salt-wasting form who are not identified by neonatal screening present with failure to thrive, dehydration, hyponatremia, and hyperkalemia typically at 7 to 14 days of life. Clinical findings of adrenal insufficiency occurred on the 4th day in this patient. Rapid progression to the salt-wasting crisis suggested a severe enzyme deficiency in the adrenal steroidogenesis pathway. Early onset of salt waste before the second week of life is a rare condition. This individual difference could also be due to varying sensitivity to aldosterone action in newborn infants. In the distal nephron, aldosterone, by binding to its receptor, tightly regulates the expression and the activity of several transport proteins implicated in sodium, potassium, and water homeostasis. A possible role of a partial and transient tubular unresponsiveness to mineralocorticoids, which is determined by genetic factors, might have effects on the timing of salt waste.

Approximately 95% of all disease-causing mutations in CYP21A2 gene are large deletions, large conversions, or one of eight point mutations (p.P30L, IVS2–13 C>G in intron 2 splice site (IVS-2), 8 bp deletion in exon 3, p.I172L, exon 6 cluster (p.I236N, p.V237E, and p.M239K), p.V281L, p.Q318X, and p.R356W) [4]. Large deletions or large conversions are typically associated with classical salt waste. This patient had a large homozygous deletion (P30L-I2G-8 bp del) in the CYP21 gene, causing severe 21OHD.

More than 90% of patients with 46, XX CAH assigned female in infancy. Evidence supports the current recommendation to raise virilised 46, XX infants with CAH as females [5]. There are no randomized controlled studies of either the best age or the best methods for feminizing surgery. Clitoral and perineal reconstruction are suggested in infancy for severely virilised (Prader stage 3) females [6]. However, an early surgery could not be performed in this patient, because of her neurogenic bladder and urinary tract infections. Meanwhile she was receiving the hormone replacement treatment properly, which provided a significant regression in the phallic structure. Both parents were quite pleased with the appearance of the external genitalia and urgent surgical intervention was not required. Deferring surgery might be an option for these patients until adolescence; however there is no evidence that either early or late surgery better preserves sexual function. Management should include the care for optimal psychosexual adjustment and increased quality of life.

Growth retardation of this patient was thought to be related to undernutrition due to frequent urinary tract infections. It was significant especially between 7th and 11th months, when she needed recurrent hospitalization. In this period hydrocortisone was used in double doses frequently, because of fever and other findings of infection. Fortunately, there was no cushingoid appearance or excessive weight gain.

This case report mainly aimed to attract attention to a concurring malformation, which might complicate the management of CAH. MMC leading to bladder dysfunction caused recurrent infections. This brought difficulties in achieving the optimal dose of glucocorticoid replacement. There are a few publications reporting congenital abnormalities concurring with CAH. Nabhan and Eugster determined upper-tract genitourinary malformations in 14 of 107 patients with CAH [7]. MMC is a common central nervous system birth defect. Various congenital and acquired abnormalities have been reported with MMC. A retrospective study [Baradaran et al.] was performed using the records of 390 patients with MMC. 17 cases of MMC with concurring congenital abnormalities, such as cardiac, musculoskeletal, and urological anomalies, were determined. Two of them had congenital adrenal hyperplasia patients with ambiguous genitalia [8]. So neural tube defects should be considered as a concurring abnormality in patients with CAH. This is one more reason why optimal care for children with CAH requires an experienced multidisciplinary team.

In conclusion, avoidance of a life-threatening crisis of adrenal insufficiency and maintaining normal height velocity and achieving normal adult height are important treatment goals in children with CAH. Long-term follow-up should include the regular monitoring of growth and bone age. Optimal health care requires monitoring of patients for signs of glucocorticoid excess as well as for signs of inadequate androgen suppression. This should remind us that management of CAH may become complicated by associating conditions.

## Figures and Tables

**Table 1:** Hormone profile of the patient before starting treatment.

Hormone	Result	Reference range	SI
**Renin**	**63 pg/mL**	3–33	**90.7 ** **µ** **U**/**m** **L**
Aldosterone	300** **pg/mL	70–1840	**30 ng/dL**
**ACTH **	**>2000 pg/mL**	10–60	**>444 pmol/L**
Cortisol	14.7** **pg/mL	4–20	406** **nmol/L
17-OHP** (LC-MS/MS) **	**50.1 ng/mL**	0.07–0.77	**151.5 ng/mL**
**Androstenedione**	**>10 ng/mL **	0.2–2.9	**>3.5 pmol/L**
**Total testosterone**	**2330 ng/dL **	14–73	**807.8 pmol/mL**
**DHEA-S**	**1945 mcg/dL**	88–356	**52.9 nmol/L**

ACTH: adrenocorticotrophic hormone; 17-OHP: 17-hydroxy progesterone; DHEA-S: dehydroepiandrostenedione sulphate.
